# Hyperferritinemia and the Extent of Mucormycosis in COVID-19 Patients

**DOI:** 10.7759/cureus.20569

**Published:** 2021-12-21

**Authors:** Simple Bhadania, Neena Bhalodiya, Yashendra Sethi, Nirja Kaka, Swati Mishra, Neil Patel, Asad Ullah Wasim, Saumya S Joshi, Kenisha Shah

**Affiliations:** 1 Otorhinolaryngology, Gujarat Medical Education & Research Society (GMERS) Medical College Sola, Ahmedabad, IND; 2 Medicine, Government Doon Medical College, Dehradun, IND; 3 Medicine and Surgery, Gujarat Medical Education & Research Society (GMERS) Medical College Himmatnagar, Himmatnagar, IND; 4 Internal Medicine, Air University Fazaia Medical College, Islamabad, PAK; 5 Medicine, Gujarat Medical Education & Research Society (GMERS) Medical College Sola, Ahmedabad, IND

**Keywords:** hyperferritinemia, extent of mucormycosis, mucormycosis, covid-19, ferritin

## Abstract

Introduction

Coronavirus disease 2019 (COVID-19) disease attributed to severe acute respiratory syndrome coronavirus 2 (SARS-CoV-2) has shown associations with various fungal opportunistic infections such as mucormycosis, invasive candidiasis, and aspergillosis, which have contributed to the mortality of the disease. In India, the incidence of mucormycosis had risen rapidly during the second wave. There is ample literature demonstrating the role of iron in the pathogenesis of mucormycosis. The hyperferritinemia associated with COVID-19 may have played a significant role in promoting the invasion and extent of the fungus.

Aims and objectives

The study aimed to analyze the association between serum ferritin levels and the extent of involvement of mucormycosis in patients affected with COVID-19.

Methodology

A single-center cross-sectional study was conducted using retrospective hospital record data. G*Power statistical analysis software was used to compute the sample size of 62 (31+31). The radiological data were used to determine the extent of involvement.

Results

A statistically significant difference was seen in levels of serum ferritin (p = 0.008) between the radiologically judged two groups of the mild extent of invasion of mucormycosis (rhinosinusitis) and severe extent of invasion (rhino-orbital/cerebral mucormycosis), with a severe extent seen with the group having higher levels of serum ferritin. Severe extent of invasion was seen in 53.6% of patients with diabetes mellitus and 62.5% of patients with both diabetes and hypertension.

Conclusion

The hyperferritinemia not only presents as a marker of the systemic inflammatory process in COVID-19 but also indicates increased free iron, which thereby aids the growth and extent of involvement by the fungus (*Rhizopus oryzae*). In individuals with diabetes and hypertension, the severity was greater. Controlling catastrophic outcomes in individuals with high serum ferritin levels necessitates extra caution.

## Introduction

Coronavirus disease 2019 (COVID-19) disease attributed to severe acute respiratory syndrome coronavirus 2 (SARS-CoV-2) was declared a global epidemic by WHO in March 2020 [1]. Due to the pervasive presence of COVID-19 all over the world, various complications and opportunistic infections associated with it have been reported [2]. During the second surge of COVID-19 cases in India in May 2021, there was a steep rise in the cases of mucormycosis in COVID-positive patients, which was particularly concerning [3]. The estimated prevalence of otherwise rare mucormycosis in India has been 80 times higher than the global prevalence [4]. There is sufficient existing literature about mucormycosis surge during that time contributing to mortality and morbidity in COVID-19 patients owing to the immune dysregulation in the background of pre-existing comorbidities [2,5].

Mucormycosis is a rare potentially fatal fungal infection caused by fungi of the order *Mucorales* occurring more frequently in predisposed immunocompromised patients [6]. The most commonly isolated species is *Rhizopus oryzae* [7]. It usually begins in the nose and paranasal sinuses and it subsequently can spread to the adjacent structures such as the orbit (rhino-orbital mucormycosis) and can progressively lead to the involvement of the brain (rhino-orbital-cerebral mucormycosis; ROCM). Thus, it can be classified into isolated nasal/rhinosinusitis, rhino-orbital, and ROCM based on anatomical involvement [8]. The fatality rate of mucormycosis is 46% globally [9].

Intracranial or intraorbital spread, which most commonly occurs in COVID-19 patients, is one of the most important factors that increases fatality rate to as high as 50-80% [9,10].

It has been well documented that immune dysfunction, preferred hypoxic environment, dysglycemia, acidic medium such as diabetic ketoacidosis (DKA)/metabolic acidosis, steroid-induced immunosuppression, and raised free iron level are all favorable factors for the development of mucormycosis in COVID-19 patients [2,6,11,12]. The current literature strongly establishes the higher risk and mortality of mucormycosis in COVID-19 patients with concomitant poorly controlled diabetes mellitus (DM) especially if associated with corticosteroid use [5,13].

As a part of the treatment protocol of severe COVID-19, corticosteroids are a mainstay [14]. The well-known adverse effects of generous usage of corticosteroids are hyperglycemic disturbances. Besides, it suppresses the function of the immune system and renders the host susceptible to infection. Both hyperglycemia and injudicious use of corticosteroids are established risk factors for the development of mucormycosis [11]. It is, however, noteworthy that DM has had a high prevalence in India with plenty of uncontrolled, undiagnosed diabetic cases even before the pandemic [15]. Furthermore, glucocorticoids have been in use in much higher doses and for longer durations in various medical conditions [10]. Mucormycosis has not been a menace in these cases. Hence, a complex interplay of several factors contributes to the development as well as more severe extents of the infection in previous or concurrent COVID-19 infected patients [5]. Thus, although DM and steroid-associated hyperglycemia are cynosures of our current understanding of the risk factors of mucormycosis, a deeper understanding beyond this is integral in view of the above facts.

Ferritin is an iron-binding protein in serum and also an acute phase reactant that is used to predict severity in COVID-19 and is commonly measured as a part of routine COVID-19 work-up [16,17]. There is ample literature demonstrating the role of iron in the pathogenesis of mucormycosis [6,18,19]. Iron is required for the growth of *R. oryzae*, as demonstrated by their lack of growth in the absence of available iron [20,21]. *Rhizopus oryzae* utilizes glucose-regulated protein 78 (GRP78) to invade endothelial cells, although it is not involved in the adherence to the cell. Hyperferritinemia and hyperglycemia have both have been implicated in the up-regulation of GRP78 during studies to prove the preponderance of mucormycosis in DKA patients [12,22]. To compound that it has been identified that SARS-CoV-2 infection has also caused an up-regulation of GRP78 [9].

This study aims to analyze and strengthen our current understanding of mucormycosis in COVID-19 by finding an association between serum ferritin and the extent of invasion of mucormycosis.

Rationale

This study aims to aid in delineating protocols for triaging the patients in the event of another surge in mucormycosis, which warrants increased suspicion for a deeper extent of involvement and extra care required in this subset of patients, thus preventing mortality associated with more severe outcomes. Despite established knowledge available on hyperferritinemia and mucormycosis, no research so far has investigated an association between levels of serum ferritin and the extent of mucormycosis, which thereby forms a potential gap in research. There is ample literature suggesting that hyperferritinemia plays a role in the severity of fungal infections.

## Materials and methods

A single-center cross-sectional study was conducted at a tertiary level hospital in Ahmedabad among the COVID-19 patients with mucormycosis admitted from May 2021 to August 2021. A sample size of 62 was calculated using G*Power statistical analysis software considering an effect size of 0.85, alpha error probability of 0.05, power of 0.95, and allocation ratio of N2/N1 of 1, dividing into two groups on basis of the extent of invasion, having a sample of 31 each representing mild invasion (rhinosinusitis) and severe invasion (orbital or cerebral invasion). The extent was judged through findings of magnetic resonance imaging (MRI) reports. All the patients included in this study had positive biopsy results and had typical MRI and computed tomography (CT) radiological features suggestive of mucormycosis. In this study, we preferred MRI over CT to appreciate the extension in orbits and the brain.

Retrospective data from the time of patient admission were used for the study. Statistical procedures were performed using SPSS Version 22.0 (IBM Corp., Armonk, NY). For discrete variables, the number of patients and percentages were given. Normality of distribution was tested using the Shapiro-Wilk test, and the Mann-Whitney U test was used to analyze the difference of serum ferritin in two groups.

Inclusion criteria

All reverse transcription polymerase chain reaction (RT-PCR) positive COVID-19 patients who were between 18 and 70 years of age having a biopsy positive for mucormycosis were included.

Exclusion criteria

Patients with a negative biopsy result or those lacking a precise biochemical or radiological record needed to assess the degree of invasion of mucormycosis were excluded.

This study was approved by the Institutional Ethical Committee of Gujarat Medical Education & Research Society (GMERS) Medical College and Civil Hospital, Sola, Ahmedabad, Gujarat (GMERSMCS/IEC/31/2021).

## Results

Our study had a total sample size of 62, which included 21 females and 41 males with a mean age of 53 years.

The difference in serum ferritin levels between patients with rhinosinusitis and patients having further invasion (rhino-orbital or rhino-cerebral) was statistically significant, as evidenced by the u-value of 436 and the p-value of 0.008. Serum D-dimer, on the other hand, has been shown to be inconsequential (Table 1).

**Table 1:** Association between serum ferritin, D-dimer, and the depth of invasion of mucormycosis in COVID-19 patients (using the Mann-Whitney U test) IQR, interquartile range

Extent	No. of patients	Serum ferritin (ng/mL)			Serum D-dimer (ng/mL)		
		Median (IQR)	u-Value	p-Value	Median (IQR)	u-Value	p-Value
Rhinosinusitis	31	193.6 (136.32-300.17)	436	0.008	749 (420-1,401.5)	293	0.531
Rhino-orbital/rhino-cerebral	31	342.1 (179.58-609.33)			541 (446-1,051)		

We were able to deduce from the data that 53.6% of patients with DM and 62.5% of patients with both DM and hypertension had a rhino-orbital or rhino-cerebral extent of mucormycosis infection, demonstrating that pre-existing comorbidity can lead to more severe infection (Table 2).

**Table 2:** Relationship between the extent of fungal involvement with different comorbidities (including DM, hypertension, or both) DM, diabetes mellitus

Comorbidities	Rhinosinusitis, n (%)	Rhino-orbital/rhino-cerebral, n (%)
None	9 (64)	5 (35.7)
DM	13 (46.4)	15 (53.6)
Hypertension	3 (75)	1 (25)
Both	6 (37.5)	10 (62.5)

Most cases with severe extent (rhino-orbital or rhino-cerebral) of mucormycosis were seen in those between 41 and 60 years of age (Table 3).

**Table 3:** Extent of mucormycosis in COVID-19 patients as per their age

Age, years	Rhinosinusitis, n (%)	Rhino-orbital/rhino-cerebral, n (%)
<40	5 (16.1)	6 (19.4)
41-60	19 (61.3)	16 (51.6)
>60	7 (22.6)	9 (29)

Among the patients with DM, the serum ferritin levels were not significantly different (p-value = 0.194) in the two groups: rhinosinusitis and rhino-orbital/rhino-cerebral (Table 4).

**Table 4:** Serum ferritin levels and extent of invasion of mucormycosis in the diabetic(s) group using the Mann-Whitney U test IQR, interquartile range

Extent	No. of patients with diabetes mellitus	Serum ferritin (ng/mL)		
		Median (IQR)	u-Value	p-Value
Rhino-sinusitis	19	285 (144.4-375.6)	182	0.194
Rhino-orbital/rhino-cerebral	25	342.1 (170.63-609.33)		

## Discussion

Mucormycosis is special being an angioinvasive infection producing tissue necrosis and infarction of the blood vessels [7]. It is caused by saprophytic fungi that belong to the order *Mucorales* [23,24]. The recent COVID-19 pandemic in the second wave saw the clustering of cases of mucormycosis in various regions of the world especially India, given that India had 70 times higher cases than the world even before COVID-19 [25]. A catena of research laid out that various factors such as steroid-induced immunosuppression, hyperglycemia, acidic medium like DKA or metabolic acidosis, and raised free iron level contributed to the development of severe mucormycosis in COVID-19 patients [2,6,11,12]. Iron plays an important role in the functioning and growth of all living beings especially fungi, and thus it is integral in the pathogenesis of mucormycosis as well [6,18,19]. The hyperferritinemia associated with COVID-19 thereby may have played a significant role in promoting the invasion and extent of the fungus.

Our study found out that most cases of severe extent (rhino-orbital or rhino-cerebral) of mucormycosis were seen in those between 41 and 60 years of age, as can be seen in Table 3, projecting it as a vulnerable age group, and this can probably be explained on the basis that this group was most exposed to environmental exposure and was among the most affected by COVID-19.

The results also indicated that a statistically significant difference was seen in levels of serum ferritin between the two groups, patients with a mild extent of mucormycosis and those with a severe extent of mucormycosis, as can be seen in Table 1. Higher levels of ferritin were associated with a severe extent. This can be explained on the basis that mucormycosis needs iron for its survival and hyperferritinemia in COVID-19, allowing free iron helps with the process of development and invasion of mucormycosis. This has been laid partly in findings of Cavezzi et al. and Sonnweber et al., who reported the role of COVID-19 interacting with the hemoglobin molecule and causing the dissociation of iron from heme molecules, which leads to hyperferritinemia in COVID-19 patients. Hyperferritinemia alters iron homeostasis, leading to iron overload in the body and thus favoring the growth and extent of mucormycosis [26,27].

No great variation was found between the extent of mucormycosis and levels of D-dimer among the two groups, which can indicate the lack of a direct relation between the two, as can be seen in Table 1. But, indeed, it is a significant inflammatory marker contributing to the disease process. D-dimer is a fibrin degradation product, assays of which are commonly used in clinical practice to exclude deep venous thrombosis and pulmonary embolism. Elevated levels of D-dimer are also seen in cancer, surgery, pregnancy, and other inflammatory states. Increased serum ferritin levels, IL-6, and D-dimer have been associated with high mortality in COVID-19 patients [28].

We also found out that among the patients with comorbidities were also severely affected; 53.57% of patients with DM as comorbidity had a severe extension of mucormycosis, whereas among patients who had both hypertension and DM, 62.5% had severe extension (rhino-orbital or rhino-cerebral involvement), as can be seen in Table 2. This can be explained based on findings of Lecube et al., who found that patients with DM showed increased serum ferritin levels, leading to increased iron stores [29]. Also, Artis et al. reported that iron and glucose both enhance the susceptibility of endothelial cells to *R. oryzae* invasion and damage by inducing over-expression of GRP78, wherein the effect of iron was more drastic. Acidosis also causes the release of free iron from iron-binding proteins [20]. Ibrahim et al. and Stanford and Voigt also supported the fact that in metabolic disorders such as DKA, the release of free iron in the blood increases, impairing iron homeostasis [6,30]. Also, in the patients with DM, the serum ferritin levels were not significantly different (p = 0.194) in the two groups (rhinosinusitis and rhino-orbital/rhino-cerebral), which indicates that the difference was not influenced by the comorbidity (Table 4). Both hyperglycemia and COVID-19 cause hyperferritinemia, and the high ferritin inside the cells cause the release of oxygen-free radicals, which damages the tissue and releases free iron in the blood. The free iron in the blood favors Mucorales growth and invasion of blood vessels, causing vessel thrombosis and tissue necrosis. Thereby, the increased extent among COVID-19 patients with DM can be explained as a product of the above-stated processes that form the fuel to help the severity of disease and extent of mucormycosis leading to poor outcomes.

Our study has certain limitations. First, it is a single-center study with a small sample size, which could restrict the generalizability of results. Second, there was a lack of histopathological grading to further define the extent of invasion.

Despite the above limitations, this study can help add data to identify patients with mucormycosis at a higher risk and thus prevent catastrophic consequences.

## Conclusions

The purpose of the study was to elicit the association between biomarkers (such as serum ferritin and D-dimer) and the extent of invasion of mucormycosis in COVID-19 patients. It was observed in our study that cases of severe extent (rhino-orbital or rhino-cerebral) of mucormycosis belonged mostly to the age group of 41-60 years. A statistically significant difference was seen in levels of serum ferritin (p = 0.008) between the two groups, with a severe extent seen in the group having higher levels of serum ferritin. Therefore, hyperferritinemia not only presents as a marker of the systemic inflammatory process in COVID-19 infection but also indicates increased free iron, which thereby aids the growth and extent of involvement by the fungus (mucormycosis). DM and hypertension were seen to act as potent aggravating factors to the disease. Special care must be taken to control severe outcomes in patients with higher levels of serum ferritin especially in people with comorbidities. Future studies are warranted to explore the role of serum ferritin and other markers of iron metabolism in the severity of mucormycosis and even other fungal diseases.
